# Common genetic variants modify disease risk and clinical presentation in monogenic diabetes

**DOI:** 10.1038/s42255-025-01372-0

**Published:** 2025-09-09

**Authors:** Jacques Murray Leech, Robin N. Beaumont, Ankit M. Arni, V. Kartik Chundru, Luke N. Sharp, Kevin Colclough, Andrew T. Hattersley, Michael N. Weedon, Kashyap A. Patel

**Affiliations:** 1 Department of Clinical and Biomedical Sciences, Faculty of Health and Life Sciences, University of Exeter, Exeter, UK; 2 Exeter Genomics Laboratory, Royal Devon University Healthcare NHS Foundation Trust, Exeter, UK

**Keywords:** Genetics, Metabolism, Endocrine system and metabolic diseases, Genomics, Diabetes

## Abstract

Young-onset monogenic disorders often show variable penetrance, yet the underlying causes remain poorly understood. Uncovering these influences could reveal new biological mechanisms and enhance risk prediction for monogenic diseases. Here we show that polygenic background substantially shapes the clinical presentation of maturity-onset diabetes of the young (MODY), a common monogenic form of diabetes that typically presents in adolescence or early adulthood. We find strong enrichment of type 2 diabetes (T2D) polygenic risk, but not type 1 diabetes risk, in genetically confirmed MODY cases (*n* = 1,462). This T2D polygenic burden, primarily through beta-cell dysfunction pathways, is strongly associated with earlier age of diagnosis and increased diabetes severity. Common genetic variants collectively account for 24% (*P* < 0.0001) of the phenotypic variability. Using a large population cohort (*n* = 424,553), we demonstrate that T2D polygenic burden substantially modifies diabetes onset in individuals with pathogenic variants, with diabetes risk ranging from 11% to 81%. Finally, we show that individuals with MODY-like phenotypes (*n* = 300) without a causal variant have elevated polygenic burden for T2D and related traits, representing potential polygenic phenocopies. These findings reveal substantial influence of common genetic variation in shaping the clinical presentation of early-onset monogenic disorders. Incorporating these may improve risk estimates for individuals carrying pathogenic variants.

## Abstract (web summary)

In clinical and population-based cohorts, a strong contribution of polygenic risk for type 2 diabetes (T2D) significantly modifies the onset and phenotypic variability of maturity-onset diabetes of the young (MODY). This polygenic T2D burden may also account for MODY-like individuals without identified monogenic causes.

## Main

Growing evidence suggests that factors beyond the primary mutation play a greater role in rare monogenic conditions than previously recognized^1,2^. Monogenic diseases are classically defined by single, highly penetrant (proportion of carriers who develop the disease) causative mutations. However, many individuals carrying the same pathogenic variant show wide variation in disease expression, suggesting that additional factors influence disease risk^3^. For example, our analysis of pathogenic *HNF1A* mutations, typically associated with young-onset diabetes, revealed striking differences in penetrance: over 90% in clinically ascertained cohorts versus under 30% in population-based cohorts by the age of 40 years^4^. This pattern of unexpectedly low and variable penetrance has now been documented across multiple monogenic conditions^5,6^. Polygenic background has been proposed as one possible contributor, particularly in age-dependent monogenic disorders^7,8^.

Maturity-onset diabetes of the young (MODY) serves as an excellent genetic disease model to investigate how common genetic variants influence young-onset monogenic disease. MODY is the most common autosomal dominant form of monogenic diabetes contributing up to 3% of all diabetes under the age of 30 years^9^. In this study we focused on the *HNF1A*, *HNF4A* and *HNF1B* genes (collectively referred to as HNF-MODY). The pathogenic variants in these three genes account for >90% of MODY cases^10–12^. These variants cause beta-cell dysfunction leading to age-dependent diabetes typically presenting before age 25 years^11^. The availability of extensive genome-wide association data for both type 1 and type 2 diabetes and related metabolic traits, widely measured diabetes markers such as HbA1c allowing accurate diagnosis, and the availability of large MODY patient cohorts make MODY particularly suitable for studying common and rare disease interplay. Together, these resources provide a robust framework for examining how polygenic factors interact with young-onset monogenic disorders.

Understanding these interactions is crucial both biologically and clinically. It can uncover new biological pathways and enhance disease prediction, knowledge that is essential for family counselling. This becomes increasingly important as genomic screening extends to clinically unselected cases and healthy newborns^13^. Previous studies have demonstrated that polygenic background can modify the penetrance of various monogenic conditions, including familial hypercholesterolaemia, obesity, kidney disease and long QT syndrome^7,14–16^. These studies are important but often lack a defined age of disease onset. Previous studies suggested that polygenic risk for type 2 diabetes (T2D) may influence the age at MODY diagnosis^17,18^. However, those studies used small sample sizes (*n* < 410), focused only on HNF1A-MODY, and did not assess diabetes-related metabolic traits or partition T2D polygenic scores to explore the underlying mechanisms in detail. A more recent study investigated the interaction between T2D polygenic risk and rare intermediate-effect variants in *HNF1A* and *HNF4A* within population cohorts but did not include clinically confirmed MODY cases with pathogenic variants^19^. No previous work has comprehensively analysed how common genetic backgrounds influence diabetes severity in MODY or quantified their overall contribution to the MODY phenotype. Finally, it remains unclear whether the common genetic background also contributes to MODY-like cases without pathogenic variants.

In this study, we investigated the interplay between polygenic background and age-dependent monogenic disorders, using MODY as a model disease. In the largest MODY cohort studied to date, we demonstrate that common genetic variants explain a substantial proportion of phenotypic variation, disease expression and may explain MODY-like phenotypes in individuals without identified monogenic causes.

## Results

### Polygenic burden of type 2 diabetes is significantly enriched in genetically confirmed MODY

**Fig. 1:**
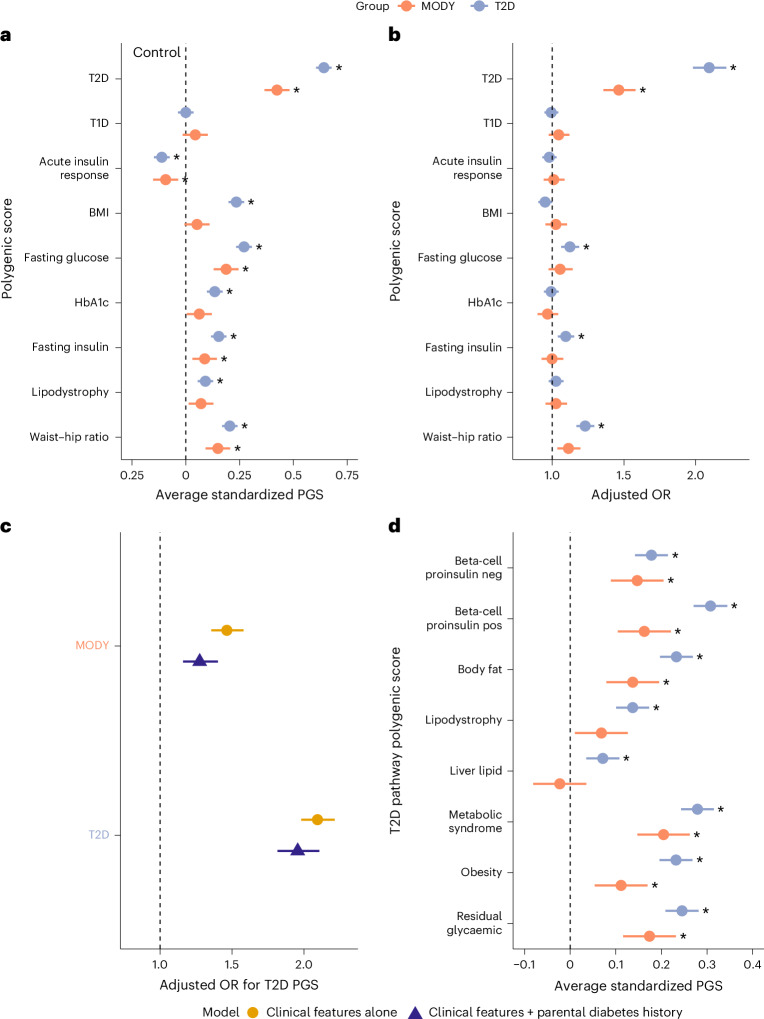
Elevated polygenic risk in HNF monogenic diabetes. **a**, Standardized differences in upper-level diabetes-related polygenic scores, determined by linear regression adjusting for the first ten within-cohort principal components. HNF-MODY carriers (orange, *n* = 1,462) and T2D cases (blue, *n* = 4,773) are compared with controls (dashed black line, *n* = 7,645). **b**, Adjusted ORs for T2D and HNF-MODY cases versus controls, assessed using a logistic regression model including each PGS, sex, age, BMI and the first ten within-cohort principal components as covariates. **c**, Adjusted ORs for T2D PGS enrichment in HNF-MODY and T2D cases under two models: (1) adjusting for covariates as described in **b** (yellow); and (2) adjusting for the same covariates plus family history of diabetes (blue). **d**, Standardized differences in T2D hard cluster partitioned polygenic scores. All scores are standardized to have a mean of 0 and s.d. of 1 in controls. ORs represent the change in risk associated with a 1 s.d. increase in the respective polygenic score. Error bars represent 95% CIs. Asterisks denote Bonferroni-adjusted statistically significant differences from controls. Sample sizes are consistent across **a**–**d**. Source data

**Extended Data Fig. 1:**
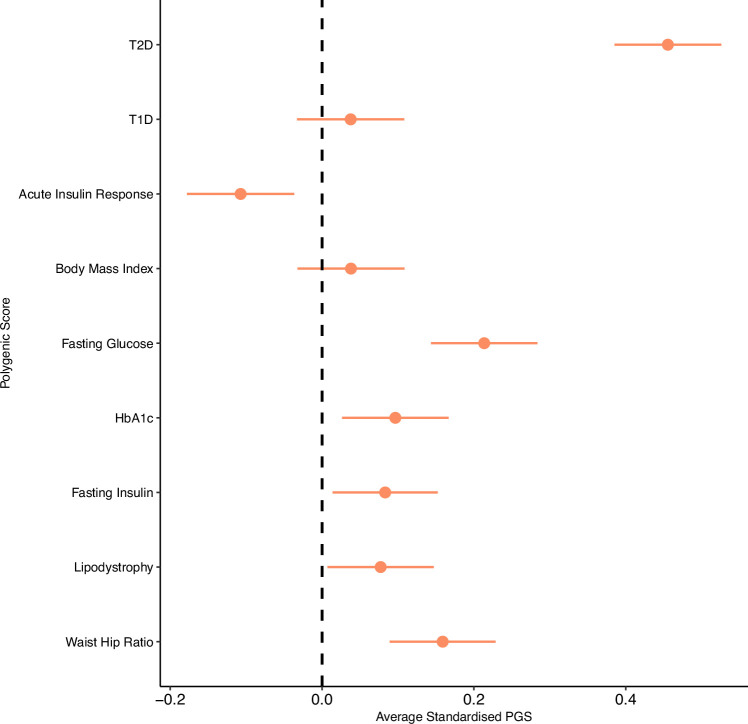
Polygenic Score difference from controls restricted to probands only. Mean polygenic score difference for MODY probands (orange, N = 924) compared to controls (dashed black line, N = 7645). Scores are standardized, with controls set to a mean of 0 and a standard deviation of 1. Error bars indicate 95% confidence intervals, and dots represent the mean estimates, determined by linear regression models adjusted for the first ten within-cohort principal components. Source data

**Extended Data Fig. 2:**
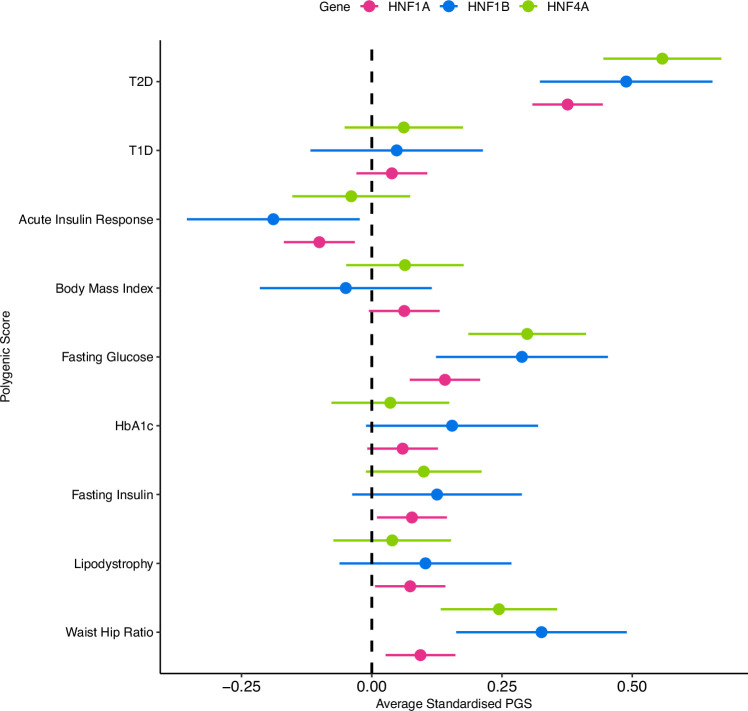
Polygenic Score difference from controls by HNF MODY subtype. Mean polygenic score differences for HNF1A (pink, N = 997), HNF1B (blue, N = 145), and HNF4A (green, N = 320) carriers compared to controls (dashed black line, N = 7645), as determined by linear regression models adjusted for the first ten within-cohort principal components. Scores are standardized, with controls set to a mean of 0 and a standard deviation of 1. Error bars represent 95% confidence intervals, and dots denote the mean estimates. Source data

**Extended Data Fig. 3:**
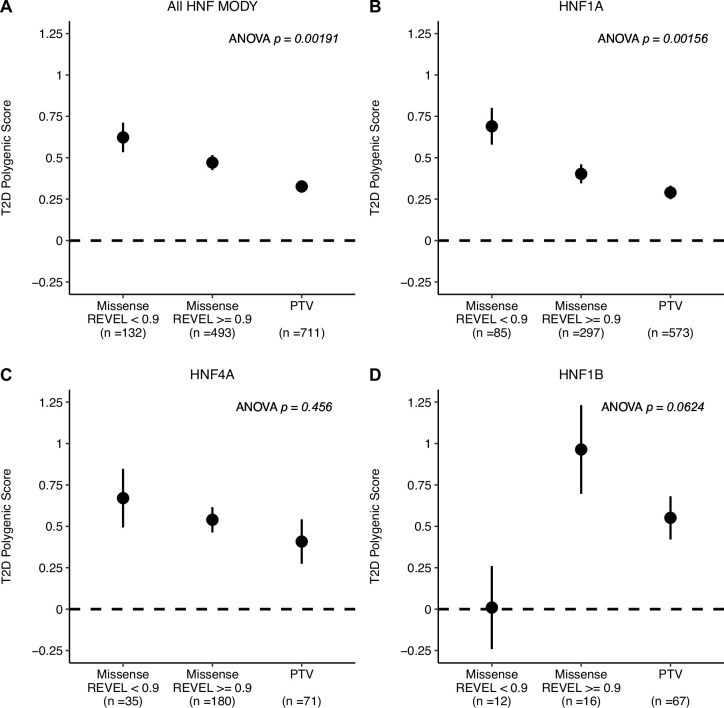
Type 2 Polygenic Risk across Mutation Severity in HNF-MODY. The graph shows mean type 2 diabetes (T2D) polygenic risk scores with 95% confidence intervals across mutation groups ordered by predicted deleteriousness, from low to high. Panel (**a**) includes all HNF-MODY genes assessed, while panels (**b**), (**c**), and (**d**) focus on *HNF1A*, *HNF4A*, and *HNF1B*, respectively. Scores are standardized, with controls set to a mean of 0 and a standard deviation of 1. We assessed statistical significance using one-way ANOVA. Source data

While common variants are known to modify disease expression in other monogenic disorders, their influence on HNF-MODY remains relatively unexplored. We investigated this assumption by analysing polygenic scores (PGSs) for T2D, type 1 diabetes (T1D) and related metabolic traits (*n* = 9) in 1,462 clinically referred patients with HNF-MODY (Supplementary Tables 1–3). We compared these scores with those of 7,645 individuals without diabetes and 4,773 individuals with T2D (Supplementary Table 1). We found significantly higher polygenic scores for T2D, fasting glucose, fasting insulin and waist–hip ratio in HNF-MODY patients compared with non-diabetic controls (0.09–0.42 s.d. increase, all *P* < 0.005) but no enrichment for T1D PGS (Fig. 1a). The T2D PGS remained the strongest contributor (odds ratio (OR) 1.46, 95% confidence interval (CI) 1.36–1.58, *P* < 0.0001) after accounting for other PGSs (Fig. 1b). This enrichment was lower than observed in T2D cases and independent of parental diabetes history (Fig. 1c) and after removing variants in *HNF1A*, *HNF4A* or *HNF1B* genes from the PGS (0.4 s.d. higher than control, 95% CI 0.35–0.46, *P* < 0.0001). To identify which T2D pathways contributed to this enrichment, we analysed eight recently developed T2D pathway-specific hard cluster PGSs^20^ (Fig. 1d). Of these, the metabolic syndrome (0.20 s.d. increase), residual glycaemic (0.17 s.d.) and beta-cell proinsulin-positive (0.16 s.d.) pathway scores showed the strongest enrichments in patients with MODY (all *P* < 4 × 10^−8^). Sensitivity analyses by each gene and limited to probands showed consistent findings (Extended Data Figs. 1 and 2). Supporting substantial interplay between rare and common variation, we observed an interaction between T2D PGS and pathogenic variants across the spectrum of predicted deleteriousness. Carriers of less-damaging missense variants showed relatively higher T2D polygenic risk compared with carriers of more deleterious protein-truncating variants (Extended Data Fig. 3). Together, these data suggest that common T2D-associated variants contribute substantially to clinically diagnosed HNF-MODY.

### Increased type 2 diabetes polygenic burden was associated with an earlier onset and greater phenotypic severity in patients with genetically confirmed MODY

**Fig. 2:**
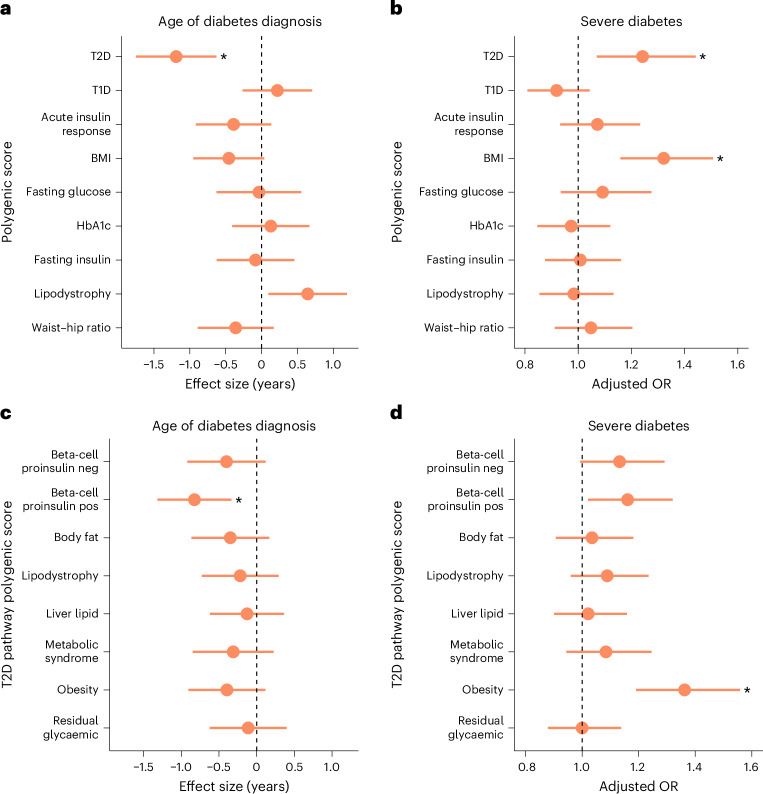
Increased polygenic risk associated with earlier and more severe diabetes diagnosis in HNF-MODY. **a**, Association between polygenic scores for upper-level diabetes-related traits and age of diabetes diagnosis. Estimates were derived using a mixed-effects linear model with family as a random effect and adjusted for other polygenic scores and the first ten within-cohort principal components. **b**, Association between polygenic scores and risk of severe diabetes (defined as HbA1c ≥ 8.5% or insulin treatment at recruitment), using a mixed-effects logistic model with the same covariates as in **a**. In total, 676 out of 1,462 MODY carriers met the criteria for severe diabetes. **c**,**d**, Association of T2D-partitioned risk scores with age of diabetes diagnosis (**c**) and diabetes severity (**d**), estimated using linear mixed-effects models adjusted for the first ten within-cohort principal components. Dots represent the estimates, with lines indicating 95% CIs. Asterisks highlight significant differences (*P* < 0.0056), after Bonferroni correction. Estimates represent the effect of a 1 s.d. increase in the respective polygenic score. All analyses in **a**–**d** were conducted in 1,462 MODY cases. Source data

We next assessed how the polygenic burden of T2D, T1D and related metabolic traits influenced both the age of diagnosis and severity of diabetes in patients with clinically identified HNF-MODY. We defined diabetes severity as either requiring insulin treatment or having HbA1c ≥ 8.5% as proposed previously^21^. Only T2D PGS demonstrated a significant association with age of diagnosis after adjusting for other PGSs (*P* < 3.3 × 10^−5^), with 1 s.d. increase linked with a 1.19 years (0.63–1.75) earlier diagnosis (Fig. 2a). In contrast, both the T2D and body mass index (BMI) PGSs were significantly associated with diabetes severity, with ORs of 1.24 (95% CI 1.07–1.44, *P* = 0.004) and 1.32 (95% CI 1.16–1.51, *P* < 3.1 × 10^−5^), respectively (Fig. 2b). Our pathway analysis revealed that the beta-cell proinsulin-positive pathway primarily drove the T2D PGS effect on diagnosis age (0.83 years (0.33–1.32) versus 0.67 (0.15–1.18) years for all others combined) (Fig. 2c). Whereas the obesity pathway demonstrated the strongest association with diabetes severity (OR 1.36, 1.19–1.56 versus 1.19,1.04–1.35 for all other pathways combined) (Fig. 2d). As expected, only the BMI PGS was significantly associated with measured BMI (Supplementary Table 4). Age of diagnosis and severity associations were maintained even after adjusting for clinical features and genetic aetiology. However, we observed strong effects of sex (females diagnosed 2.28 years earlier), maternal diabetes history (diagnosed 3.54 years earlier) and BMI (0.24-year earlier diagnosis) on age of diagnosis (Supplementary Tables 5 and 6). Sensitivity analyses by each gene show directional consistent results (Supplementary Table 7). We also conducted additional sensitivity analyses in 413 HNF-MODY cases with available birthweight data and found that associations between T2D polygenic scores and clinical outcomes remained largely unchanged after adjusting for birthweight (Supplementary Tables 8 and 9). These findings highlight the complex interaction between genetic and clinical factors that shape the clinical presentation of HNF-MODY.

### Type 2 diabetes polygenic burden modifies the risk of diabetes in clinically unselected carriers of pathogenic HNF-MODY variants

**Fig. 3:**
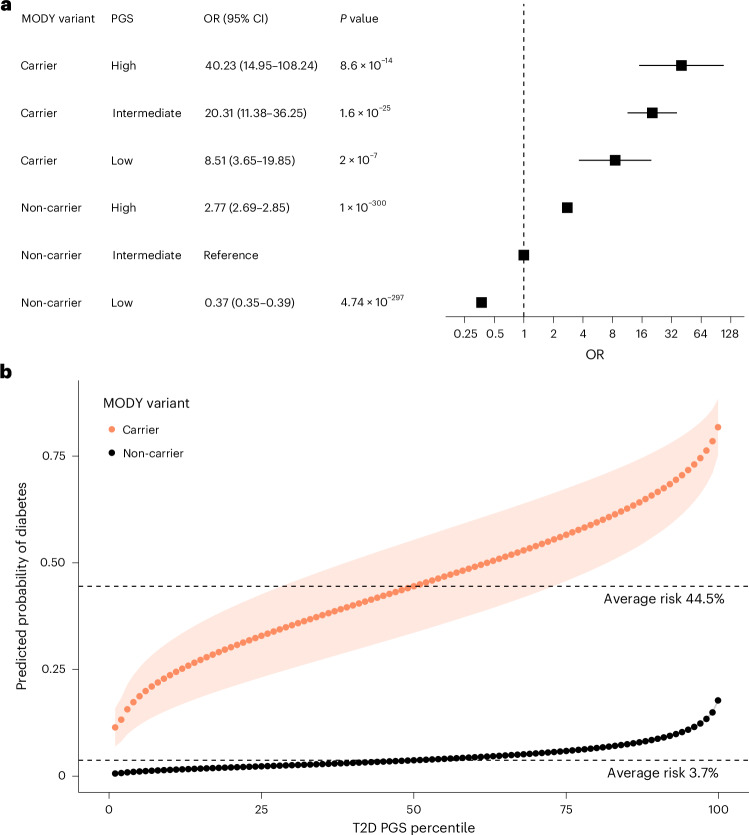
Polygenic background modifies diabetes risk in clinically unselected HNF-MODY carriers. **a**, ORs for diabetes risk in HNF-MODY carriers and non-carriers, stratified by T2D polygenic risk levels. Points represent ORs with error bars representing 95% CIs. Low risk is defined as the bottom 20% of T2D polygenic scores, high risk as the top 20% and intermediate risk as the remaining 60%. *n* = 88,905, 265,079 and 70,469 for non-carriers and *n* = 28, 55 and 17 for carriers, for low, intermediate and high T2D PGS risk, respectively. Diabetes risk was estimated using a two-sided logistic regression model adjusted for sex, age, family history of diabetes, the first ten ancestry principal components and BMI. **b**, Predicted probability of diabetes at baseline across each percentile of T2D polygenic risk, assessed using a logistic model with T2D polygenic score as a continuous variable and the same covariates as in **a**. Points represent predicted mean probability per percentile, and shaded regions represent 95% CIs. Dashed lines indicate the baseline diabetes risk at the 50th percentile of T2D polygenic scores for HNF-MODY carriers and non-carriers. Source data

We next investigated how polygenic T2D background influences diabetes risk in carriers of pathogenic HNF-MODY variants. To assess this, we needed to investigate individuals not ascertained clinically to see the clear effect of polygenic background. Therefore, we analysed 424,553 European individuals with whole exome sequencing data from the clinically unselected UK Biobank population cohort. Among these, 100 individuals were identified as carriers of pathogenic variants in *HNF1A* (*n* = 34), *HNF4A* (*n* = 51) or *HNF1B* (*n* = 15) (Supplementary Tables 10 and 11). Using a T2D PGS that did not include UK Biobank in the discovery cohort^19^, we found that among mutation carriers, diabetes risk varied substantially by T2D PGS. Compared with non-carriers with intermediate T2D PGS (middle three quintiles), carriers’ risk ranged from 8.5-fold (95% CI: 3.65–19.85) in those in the lowest T2D PGS quintile to 40.22-fold (95% CI 14.95–108.24) in those in the highest quintile (Fig. 3a). HNF-MODY carriers had a 6.67-fold higher risk of diabetes (95% CI 4.39–10.12, *P* = 4.23 × 10^−19^) compared with non-carriers in the highest quintile, highlighting the strong impact of pathogenic mutations. Despite the limited sample size, diabetes risk seemed to rise consistently across the range of T2D PGS, with diabetes risk ranging from 11.4% (first percentile, 95% CI 6.96–15.88) to 81.7% (99.9th percentile, 95% CI 75.17–88.34) (Fig. 3b). Notably, non-carriers in the 99.9th T2D PGS percentile showed a 17.7% risk (95% CI 17.3–18.2), which was similar to mutation carriers with lowest T2D PGS. A sensitivity analysis using T2D PGS, which excluded variants within 1 Mb of the three MODY genes, showed similar effect sizes (OR 2.17, 1.2–3.91 per 1 s.d. change whole PGS versus 2.06, 1.16–3.82 without MODY genes). These data together suggest a substantial contribution of T2D polygenic background on diabetes risk in HNF-MODY, while some individuals without MODY mutations but with extreme polygenic risk may reach a similar risk as HNF-MODY.

### Common genetic variants explain 24% of phenotypic variance in MODY

**Fig. 4:**
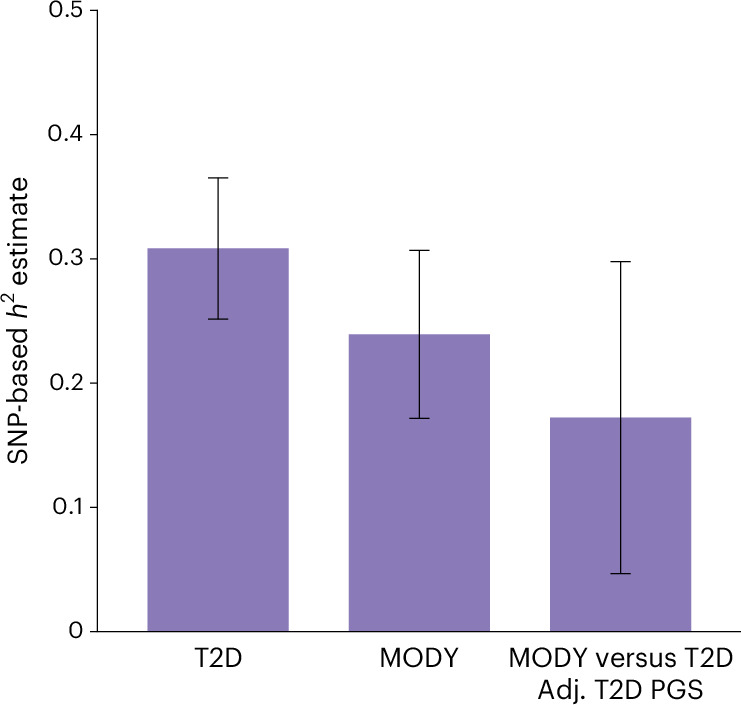
SNP-based heritability estimates for MODY. SNP-based *h*² estimates were calculated in unrelated individuals using GCTA GREML-LDMS, stratified into four LD bins of equal size to construct the genetic relationship matrix, with sex, age and the first ten principal components within the cohort as covariates. Bars represent heritability point estimates and error bars represent the 95% confidence intervals. Estimates for HNF-MODY carriers (*n* = 864) and T2D cases (*n* = 4,461) were compared with non-diabetic controls (*n* = 6,935). *h*² is shown on the liability scale for T2D (prevalence, 0.1) and MODY (prevalence, 0.0005). MODY and T2D cases were compared, adjusting for T2D polygenic score. Source data

Having observed substantial contribution of polygenic background, we next aimed to quantify the overall contribution of common genetic variants to MODY. Using genome-wide complex trait analysis (GCTA) genome-based restricted maximum likelihood (GREML), we estimated common variant (minor allele frequency > 0.01) single nucleotide polymorphism (SNP) heritability (*h*^2^), on the liability scale. Our analysis revealed a SNP heritability of 23.9% (95% CI 17.2–30.7%, *P* < 0.0001) in individuals with HNF-MODY (Fig. 4). This estimate was only slightly lower than in polygenic T2D cases 30.8% (95% CI 25.08–36.61%, *P* < 0.0001). The heritability estimate remained consistent across multiple approaches, including restricting to HNF1A-related monogenic diabetes, phenotype correlation–genotype correlation regression and applying GREML estimation in linkage-disequilibrium adjusted kinships (LDAK) (Supplementary Table 12). To determine how much of this common variant heritability stems from T2D-associated variants, we calculated SNP-heritability for MODY comparing against 4,461 T2D cases, both with and without T2D PGS adjustment. The heritability decreased to 20.3% when compared with T2D, and further dropped to 17.2% (95% CI 4.7–29.7%, *P* = 0.035) after T2D PGS adjustment (Fig. 4). These findings reveal that common genetic variants substantially influence MODY’s clinical presentation. At least one-third of this influence comes from T2D variants, suggesting the presence of T2D-independent genetic modifiers in HNF-MODY.

### Clinically referred MODY cases without a pathogenic variant have substantially higher polygenic burden of T2D and related traits

**Fig. 5:**
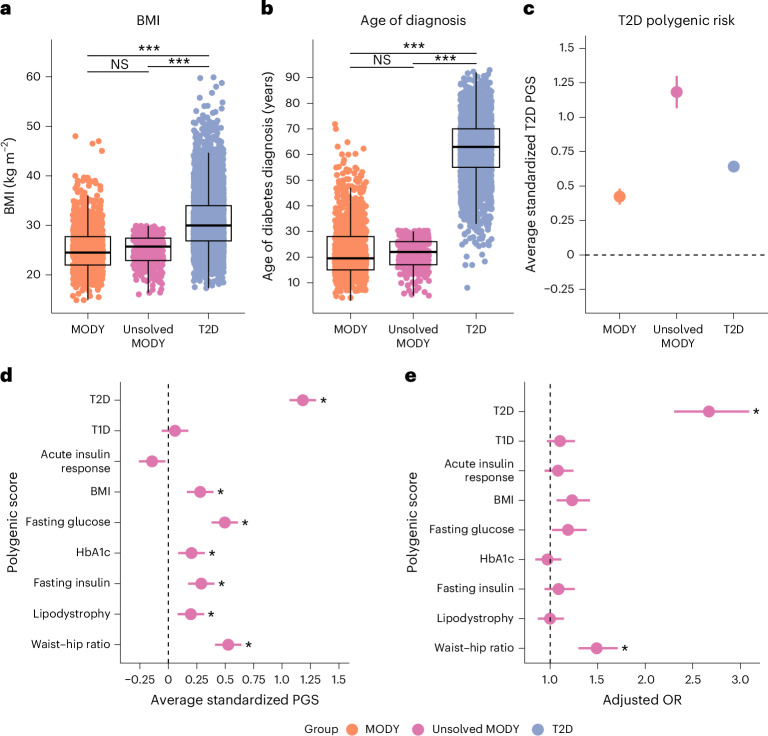
Unsolved MODY cases exhibit extreme T2D polygenic risk while phenotypically resembling typical MODY cases. Distribution of key characteristics and polygenic risk across MODY carriers (orange, *n* = 1,462), T2D cases (blue, *n* = 4,773) and unsolved MODY cases (pink, *n* = 300). **a**,**b**, Distribution of the clinical characteristics BMI (**a**) and age at diabetes diagnosis (**b**), collected at patient referral. Box plots display the median and interquartile range, with individual data points overlaid. Statistical significance between groups is indicated; ***P < 0.017; NS, not significant, as determined by two-sided *t*-tests. **c**, Mean T2D polygenic risk across groups versus controls (*n* = 7,645), assessed using a two-sided linear regression model adjusting for the first ten within-cohort principal components. **d**, Mean polygenic risk score differences for diabetes-related traits in unsolved MODY cases versus controls, using the same method as in **c**. **e**, Adjusted ORs for unsolved MODY cases versus controls, assessed using a two-sided logistic regression model including each PGS, sex, age, BMI and the first ten within-cohort principal components as covariates. In **d** and **e**, asterisks denote Bonferroni-corrected significant differences from controls (*P* < 0.0056). In **c**–**e**, dots represent point estimates, with error bars representing 95% CIs. Source data

**Extended Data Fig. 4:**
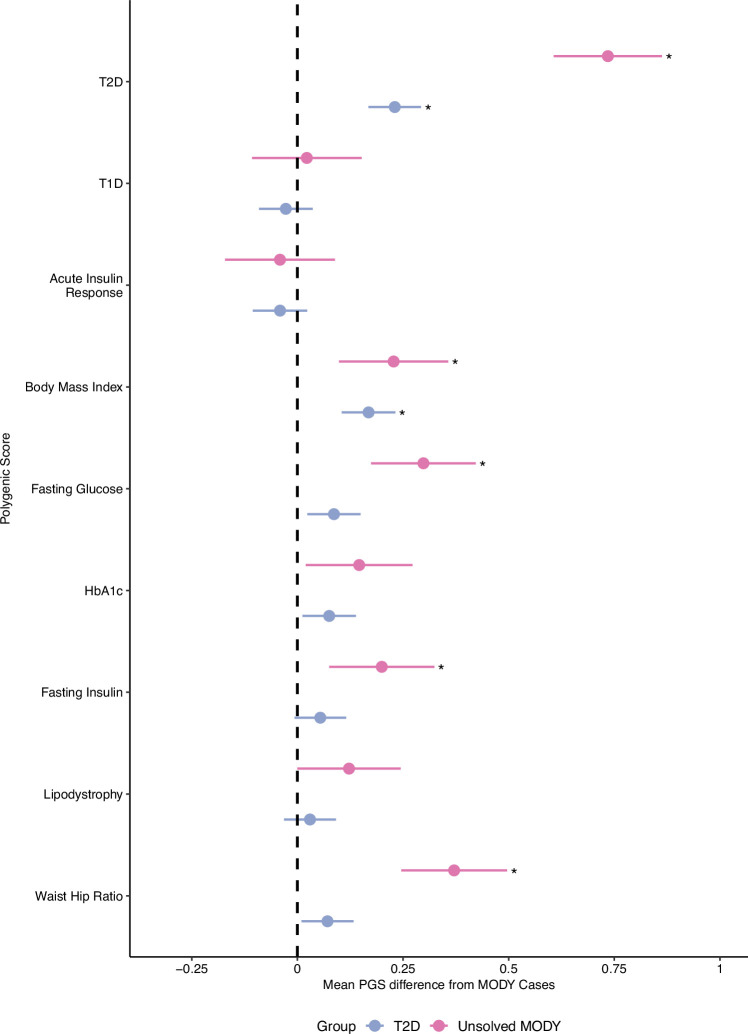
Mean polygenic score difference from solved MODY cases. Mean polygenic score difference between unsolved MODY cases (N = 300, pink) and T2D cases (N = 4773, blue) versus MODY cases (N = 1462, assessed using a logistic regression model including the first ten within-cohort principal components as covariates. Asterisks denote significant differences (P < 0.0028). Polygenic Scores were standardized so that the control population has a mean of 0 and standard deviation of 1. Error bars represent 95% confidence intervals. Source data

**Extended Data Fig. 5:**
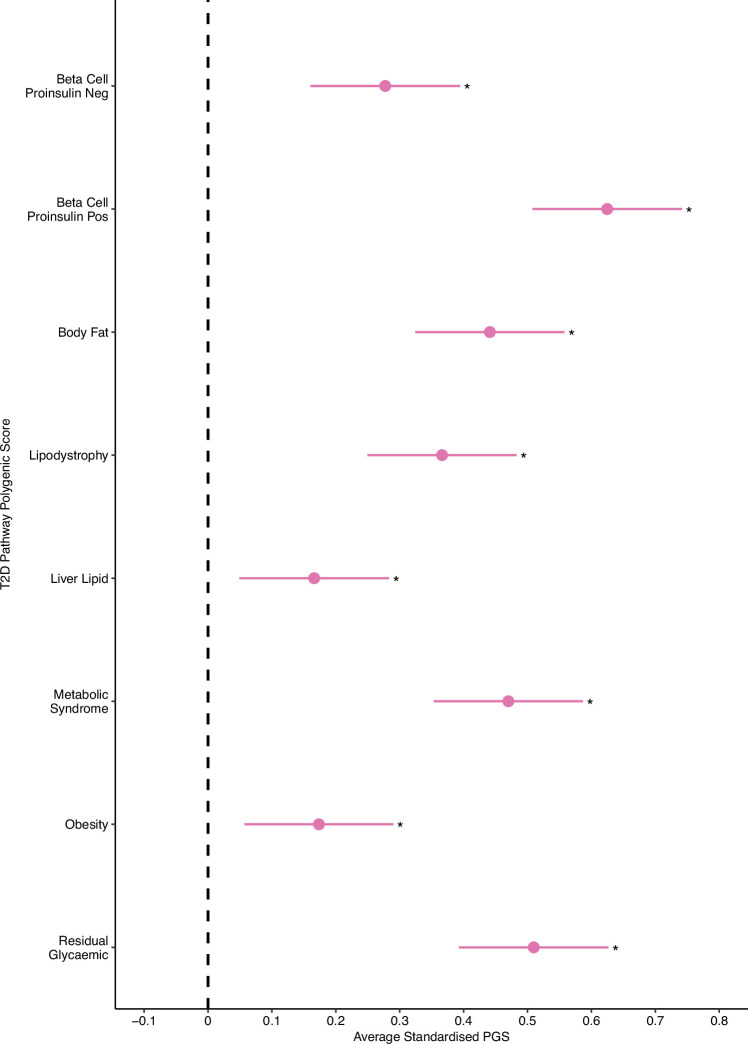
T2D Hard Cluster Polygenic Score Differences in Unsolved MODY Cases Compared to Controls. Mean polygenic score differences for T2D hard cluster partitioned scores in Unsolved MODY cases (pink, N = 300) compared to non-diabetic controls (dashed black line, N = 7,645). Scores are standardized with controls set to a mean of 0 and a standard deviation of 1. Error bars represent 95% confidence intervals, and dots indicate the mean estimates. Asterisks denote statistically significant differences from controls (P < 0.006), based on the Bonferroni significance threshold, as determined by linear regression models adjusted for the first ten within-cohort principal components. Source data

**Extended Data Fig. 6:**
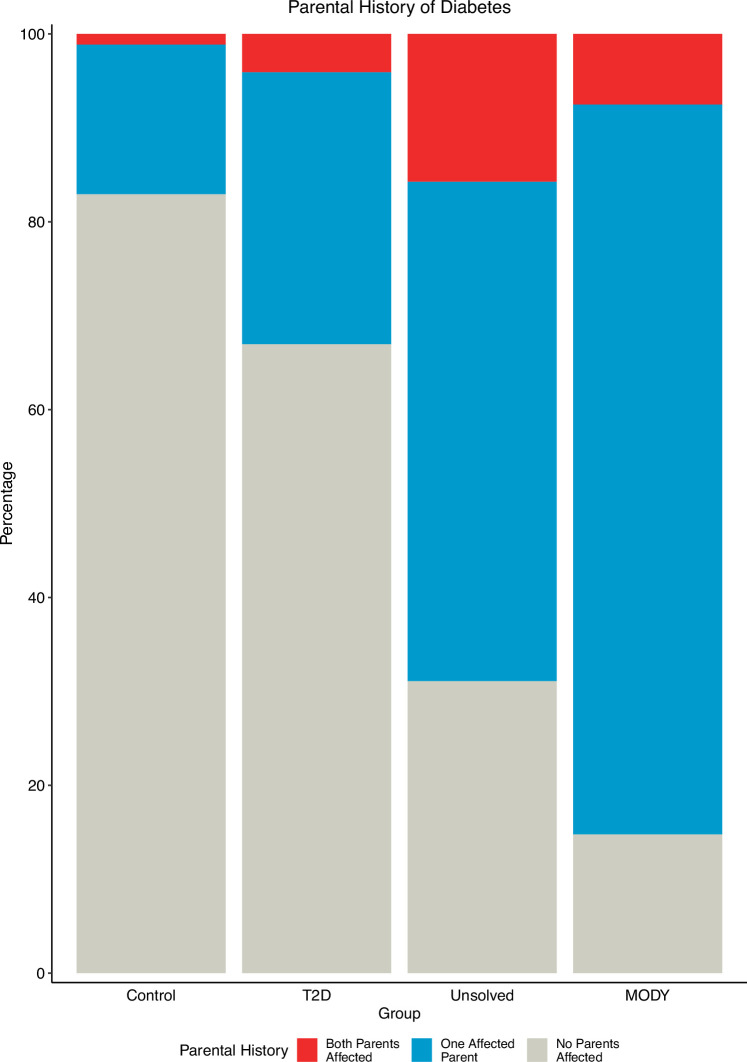
Unsolved MODY cases have a high percentage of familial diabetes history. The plot shows the distribution of parental family history of diabetes for four groups: non-diabetic controls (N = 7,645), genetically confirmed MODY cases (N = 1,462), Unsolved MODY cases (N = 300), and type 2 diabetes cases (N = 4,773). Family history is categorized into three groups: no parent with diabetes (grey), one parent with diabetes (blue), and both parents with diabetes (red). Percentages are calculated within each group. Source data

Following our observations of substantial common genetic variant contributions in patients with mutation-positive MODY, we investigated whether higher polygenic background could also explain diabetes in individuals with a MODY phenotype but without causative mutations in known monogenic diabetes genes. We studied 300 individuals referred for MODY genetic testing from routine clinical practice with diabetes diagnosis before age 30 and BMI < 30 kg m^−2^, and without evidence of T1D (positive islet autoantibodies, C-peptide <200 pmol l^−1^, or high T1D genetic risk score >50th centile of the T1D population)^22^. These unsolved MODY cases showed similar age of diagnosis and BMI to mutation-positive MODY cases (*P* > 0.05 for both) (Fig. 5a,b and Supplementary Table 13). As expected, these unsolved cases showed no excess T1D PGS but displayed a striking 1.18 s.d. (95% CI 1.07–1.29, *P* < 0.0001) higher T2D PGS than controls (Fig. 5c). This polygenic burden was higher than both mutation-positive MODY cases by 0.73 s.d. and T2D cases by 0.52 s.d. (all *P* < 0.0001) (Extended Data Fig. 4). Compared with controls, we also observed an excess polygenic burden of BMI and waist–hip ratio in these cases (Fig. 5d,e). Unsolved cases demonstrated an enrichment in all T2D partitioned PGSs, with the largest difference from controls in the beta-cell proinsulin-positive cluster (0.62 s.d. increase, 95% CI 0.51–0.74, *P* < 0.0001) (Extended Data Fig. 5). Excess biparental diabetes history further supported the observed excess polygenic enrichment in unsolved cases compared with T2D (53% one parent, 15.7% both parents with diabetes versus 28.9% and 4%, respectively in T2D) (Extended Data Figure 6). These findings suggest that while some unsolved cases may harbour novel monogenic diabetes mutations, many likely represent polygenic phenocopies driven by an excessive polygenic burden of T2D and related traits.

## Discussion

In this study, we demonstrate that HNF-MODY has a significant polygenic component, with common genetic variation substantially influencing disease onset and severity in genetically confirmed MODY cases. The elevated polygenic burden of T2D-related traits may explain MODY phenocopies lacking pathogenic mutations.

MODY’s genetic architecture seems more complex than its traditional characterization as a purely monogenic disorder. We found that common genetic variations explain approximately 24% of phenotypic variance in clinically identified cases. This estimate is substantially higher than previously reported in other monogenic disorders (long QT syndrome, 15%^16^ and developmental delay, 11% (ref. ^23^)) and approaches that of T2D. Such high polygenic contribution is unexpected for a presumed monogenic disease and may reflect its young-onset nature. T2D-associated variants had the strongest effect among the traits we analysed, likely because of shared pathways in beta-cell dysfunction. We found that T2D polygenic risk influenced age at diagnosis mainly through proinsulin-associated beta-cell pathways, which supports current understanding that HNF-MODY arises primarily from beta-cell dysfunction. Our findings on the relationship between T2D polygenic scores and age at diagnosis are consistent with smaller studies in HNF1A-MODY^17,18^.The absence of interaction with T1D polygenic risk aligns with the current understanding that T1D variants primarily affect autoimmune pathways rather than transcriptional networks disrupted in HNF-MODY^24^. This genetic distinction supports using T1D polygenic risk scores to differentiate MODY from early-onset T1D^22,25^.

Our findings reveal distinct genetic pathways modifying different aspects of MODY. Beta-cell proinsulin-related variants predominantly influence age of diagnosis, while obesity-associated variants and beta-cell pathways drive disease progression. This supports a liability threshold model where pathogenic MODY variants drive early-onset disease, with the polygenic background modifying overall disease risk. We observed that the polygenic contribution is not constant but depends on underlying pathogenic variant where less-damaging variants require more contribution for disease expression and clinical diagnosis. Importantly, we show that T2D polygenic risk strongly modifies diabetes risk in individuals carrying pathogenic MODY variants. Previous studies have reported similar effects for intermediate-effect variants in HNF1A and HNF4A in population cohorts, where common T2D risk variants altered the penetrance of those rare alleles^19^. Notably, pathogenic variant carriers with low T2D polygenic risk show substantially lower diabetes risk, with about half remaining disease-free in the population cohort. This explains the disparity between MODY prevalence in clinical referrals (1:10,000) versus genetic screening (1:2,000)^4,26^. Together, these data demonstrate that MODY’s pathogenesis involves substantial polygenic interaction rather than following a simple deterministic monogenic model.

Some unsolved MODY cases may represent polygenic phenocopies. Our small cohort of mutation-negative cases shows substantial enrichment of T2D polygenic risk exceeding that seen in typical T2D. This enrichment extends beyond T2D to other related traits, supporting complex polygenic aetiology. Similar patterns are observed in other monogenic conditions like long QT syndrome^16^, developmental delay^23^ and familial hypercholesterolaemia^14^, where mutation-negative patients show higher polygenic burden than mutation-positive cases. These unsolved cases likely represent a heterogeneous group with multiple underlying causes, including potential overlap with previously defined T2D subtypes^27,28^. Although our sample size limited detailed clustering, it is plausible that some individuals may align with distinct mechanistic pathways, as observed in these subgroups of T2D. While some may resemble the severe insulin-deficient diabetes subgroup, our findings suggest broader enrichment across all T2D risk pathways. This implies that the unsolved MODY group does not map cleanly onto existing subtypes. It likely includes individuals at the extreme tail of the polygenic risk distribution, possibly carrying rare, low-penetrance variants that act additively with high polygenic burden to drive clinical referral. Collectively, these findings suggest the presence of polygenic phenocopies. However, due to the relatively small sample size, these results should be considered as preliminary. Further studies are needed to replicate these observations and elucidate the underlying mechanisms.

Our findings support the hypothesis that monogenic disorders exist on a continuum, where both pathogenic mutations and polygenic background shape disease manifestation^29^. Age-dependent conditions, such as MODY, are likely to have a larger polygenic contribution compared with neonatal-onset disorders. As evidence accumulates, this observation may extend to the majority of monogenic disorders, albeit to varying degrees. However, each condition will require individual evaluation to quantify the relative contributions. With the declining cost of genetic testing and the increasing identification of presymptomatic carriers through incidental findings^30^ and newborn screening programmes^31^, there is a growing need to refine disease risk prediction. Currently, risk assessment relies solely on the presence of pathogenic mutations. To provide more precise risk stratification, it may be necessary to incorporate non-mutation factors, such as polygenic risk scores or family history, as is already done in conditions such as breast cancer^32^. As whole-genome sequencing moves toward becoming a first-line test, a single assessment could offer comprehensive genetic information, incorporating both monogenic and polygenic risk. However, clinical implementation will require large-scale, multi-ancestry MODY datasets and collaboration across dedicated cohorts to enable robust model development, validation and equitable application. Further research is needed to evaluate the added clinical value of this approach in improving diagnosis and risk prediction.

Although this is the largest MODY study to date, the sample size for individual genes and unsolved cases limited our power to detect subgroup-specific effects. Despite this, the direction and strength of associations were consistent across the HNF-MODY subtypes, supporting the generalizability of our findings. The predominantly UK-based, European ancestry cohorts limit generalizability to other populations. While MODY variants in the UK Biobank were not Sanger-confirmed, we minimized false positives through manual IGV review and strict quality filtering. The UK Biobank’s healthy volunteer bias likely underestimates true MODY penetrance in general populations due to underrepresentation of early-onset diabetes. Furthermore, our sample size limited more detailed analysis of non-clinically referred HNF-MODY carriers. Our local MODY cohort is derived from routine clinical referrals across the UK, so case ascertainment may be influenced by environmental factors such as healthcare access, socioeconomic status, clinical practice variability and other unmeasured confounders. We adjusted our analyses of age at diagnosis and disease severity for several known or measurable confounding factors, including variant location, family ID, proband status, BMI, parental diabetes, birthweight and year of diagnosis. However, we could not adjust for lifestyle-related factors such as diet, physical activity, early-life factors, educational attainment, social status, treatment preference or adherence. While these limitations remain, the large sample size may mitigate some of their effects. The observation of similar associations between T2D risk and age at diagnosis in multigenerational pedigrees from Finland^18^ supports the robustness of our findings and suggests that these biases important but does not explain the all the results.

In summary, using MODY as a model disease, we demonstrate substantial interplay between monogenic mutations and polygenic background in young-onset monogenic disorders. Our findings suggest that future approaches to disease prediction will require integration of monogenic, polygenic and environmental factors to improve clinical utility.

## Methods

### Study populations

This study complies with all relevant ethical regulation and was approved by the appropriate ethics committees. Our study combined three ethically approved cohorts. In our local MODY cohort, all probands or their guardians provided informed consent, and the North Wales Ethics Committee approved the study, with Genetic Beta Cell Research Bank approving sample access. The National Institute for Health Research (NIHR) Exeter Clinical Research Facility management committee approved access to these samples and genotype data for our T2D and non-diabetic controls. This research also utilized data from the UK Biobank resource carried out under UK Biobank application number 103356. UK Biobank protocols were approved by the National Research Ethics Service Committee.

### Exeter MODY cohort

#### MODY individuals with confirmed pathogenic variants

We analysed individuals referred for monogenic diabetes genetic testing at the Exeter Genomics Laboratory, Royal Devon University Healthcare NHS Foundation Trust, Exeter, UK. These referrals originated from clinical suspicion of MODY during routine clinical care in the UK. These individuals were found to have likely pathogenic or pathogenic variants either by Sanger sequencing or gene panel test performed as part of routine clinical care. Our cohort comprised European individuals with diabetes and carrying pathogenic variants in *HNF1A* (*n* = 997), *HNF1B* (*n* = 145) or *HNF4A* (*n* = 320). We focused on the more commonly diagnosed, age-dependent forms of MODY (*HNF1A*, *HNF4A* and *HNF1B*). We excluded GCK-MODY because it represents a fundamentally different disease: individuals present with lifelong, mild fasting hyperglycaemia that does not progress with age, does not require treatment and is not associated with excess complications^33^. In this context, age at diagnosis reflects the timing of detection rather than age at disease onset.

#### Unsolved MODY individuals

We evaluated 300 European individuals referred from routine clinical care in the UK with suspected MODY. All participants received their diabetes diagnosis before age 30 years and lacked clinical features suggestive of T2D (BMI ≥ 30 kg m^−2^) or T1D (positive islet autoantibodies, C-peptide <200 pmol l^−1^ and a ten-SNP T1D genetic risk score above the 50th centile of the gold-standard T1D population from the WTCCC study)^22^. These individuals underwent comprehensive genetic testing for all known monogenic diabetes genes (*n* = 58) and were not found to have pathogenic variants in these genes. The clinical features of these solved and unsolved MODY cases, at referral for genetic testing, are summarized in Supplementary Table 1.

### Type 2 diabetes and non-diabetes control cohort

We analysed participants from two ethically approved population cohorts in Southwest England: the Exeter 10000 study (https://exetercrfnihr.org/about/exeter-10000/)^34^ and the Diabetes Alliance for Research in England study (https://www.diabetesgenes.org/current-research/dare/)^35^. These studies recruited unselected participants through primary care practices across the Southwest United Kingdom. At recruitment, participants completed baseline questionnaires and provided fasting blood and urine samples for measurement of diabetes-related markers, including fasting glucose and HbA1c. Our analysis included European individuals who underwent array genotyping as part of these studies. We classified participants as having T2D if they either did not require insulin treatment or initiated insulin treatment after 36 months from diagnosis, thereby excluding potential misclassified T1D cases. We defined controls as individuals without a known diabetes diagnosis and HbA1c ≤ 48 mmol mol^−1^ (6.5%)^36^. The final cohort comprised 7,645 controls and 4,773 individuals with T2D, with their clinical characteristics presented in Supplementary Table 1.

### UK Biobank cohort

The UK Biobank represents a large-scale, prospective population-based study comprising approximately 500,000 UK residents aged 40–70 years at enrolment^37^. Recruitment occurred between 2006 and 2010, with comprehensive data collection through multiple channels: participant questionnaires, structured interviews and biomarker measurements^37^. The study supplemented this information with medical history data from Hospital Episode Statistics records coded using ICD-9 and ICD-10 codes. We defined diabetes status using three criteria: self-reported diagnosis, HbA1c levels ≥6.5 % at recruitment or active diabetes treatment at recruitment. Our study cohort consisted of 424,553 European individuals who underwent exome sequencing and array genotyping. Clinical characteristics of these individuals can be found in Supplementary Table 10. We analysed the exome sequence data to identify individuals with likely pathogenic and pathogenic variants in *HNF1A*/*HNF4A*/*HNF1B* as described previously^4^, with details of variants identified in Supplementary Table 11.

### Genetic analysis

#### MODY pathogenic variants in Exeter MODY cohort and UK Biobank

For the Exeter MODY cohort, all referred patients were screened for potential MODY-associated variants using either Sanger sequencing or gene panel testing, following the methodologies detailed by Ellard et al.^38^. For the UK Biobank participants, we utilized exome sequence data to identify carriers of pathogenic MODY variants. We annotated all variants using clinically validated transcripts: GenBank NM_000545.6 for *HNF1A*, NM_000458.4 for *HNF1B* and NM_175914.4 for *HNF4A*. We classified variants according to the American College of Medical Genetics and Genomics/Association of Molecular Pathology guidelines, designating them as either likely pathogenic (class 4) or pathogenic (class 5)^39^. This classification process followed our established protocols for the local Exeter cohort and aligned with our recent study’s methodology^4^. Supplementary Table 11 presents a comprehensive list of variants identified in the UK Biobank cohort.

#### Array genotyping

##### Exeter MODY, T2D and non-diabetic controls

We performed array genotyping using the Infinium Global Screening Array platform. Our comprehensive quality control protocol excluded samples with call rates below 98%, sex mismatches, relationship discrepancies or inbreeding coefficients exceeding 0.1. At the variant level, we removed markers with missingness above 2%, minor allele frequency below 5% or deviation from the Hardy–Weinberg equilibrium (*P* < 1 × 10^−6^). We applied these quality control measures both independently for each batch and following batch integration. We then used linkage disequilibrium (LD) pruned markers for genotype imputation through the TOPMed reference panel v.2 (ref. ^40^) via the Michigan Imputation Server^41^. To determine genetic ancestry, we compared our data with reference populations from the 1000 Genomes Phase 3 and Human Genome Diversity Project, implementing a principal component analysis (PCA) approach within the GenoPred Pipeline (v.2.2.1)^42,43^. For relationship inference, we analysed LD-pruned data using the KING robust algorithm (v.2.2.4) to identify unrelated individuals up to the third degree^44^. To better capture the within-cohort population structure, we conducted PCA using FlashPCA (v.2.0)^45^. Initially, we calculated principal components in unrelated European individuals and then projected these onto related European individuals.

##### UK Biobank

The UK Biobank individuals were SNP-genotyped using the UK BiLEVE array for the first ~50,000 individuals, with the remaining using the UK Biobank Axiom array. This dataset underwent central quality control by the UK Biobank and was imputed to the TOPMed reference panel^40^. Approximately 450,000 individuals from the UK Biobank Array also underwent exome sequencing using the IDT xGen Exome Research Panel v.1.0. Detailed sequencing methodology for UK Biobank samples has been described previously^46^. In brief, variants were called using GATK v.3.0 filtering variants with an inbreeding coefficient <−0.03 or without at least one variant genotype of DP ≥ 10, GQ ≥ 20 and, if heterozygous, AB ≥ 0.20. For this analysis, we included 424,553 individuals who had both exome and array data and were of European ancestry, inferred from projected PCA using the same approach as for the local cohort.

### Polygenic score calculation

We calculated polygenic scores for T2D^20^, T1D^47^ and seven diabetes-related traits^48–52^, alongside eight pathway-specific T2D risk scores^20^. We constructed weighted polygenic scores using genome-wide significant variants. For traits with comprehensive genome-wide association study (GWAS) summary statistics available, we implemented genome-wide calculations to capture additional genetic signal. Our computational pipeline utilized PLINK 1.9’s score function for genome-wide significant variant-based scores^53^. For the genome-wide polygenic scores, we implemented the GenoPred v.2.2.1 pipeline with the LDpred2 auto model, enabling comprehensive processing of GWAS summary statistics^43,54^. Further details are available in Supplementary Table 3, including the specific approach used for each trait, including the calculation method, number of variants incorporated and the source GWAS studies.

### Heritability estimation

To estimate the common variant contribution to MODY and T2D, SNP-based heritability was estimated in unrelated individuals using GCTA GREML-LDMS, stratifying into four LD bins of equal size to construct the genetic relationship matrix.^55^ To test the validity of these estimates we ran phenotype correlation–genotype correlation and restricted maximum likelihood approaches implemented in LDAK, using thinned predictors to construct the kinship matrix^56,57^. We used sex, age and the first ten within-cohort principal components as covariates for each method. For MODY, disease prevalence was set at 0.0005^4^ and 0.00025^26^, and for T2D, at 0.1^58^ (Table S12). Variants with an imputation quality > 0.9 and minor allele frequency > 1% were used to in this analysis.

### Statistical analysis

#### Assessing common variant enrichment in MODY cohort

To assess polygenic risk in MODY carriers and T2D cases, we employed several different approaches. To initially assess whether any common variant pathways contribute to clinically referred HNF-MODY we tested nine PGSs for enrichment. All scores were standardized using the control group as reference (mean = 0, s.d. = 1). To test differences in polygenic scores from controls, we used linear models adjusting within-cohort principal components to control for population structure. We assessed each score individually first, however, due to well-known overlaps of variants across these related metabolic traits, we then performed multivariable logistic regression analysis to identify the key independent common variant pathways contributing to HNF-MODY after adjusting for sex, age, BMI and the first ten within-cohort principal components. We repeated these steps with unsolved MODY cases to examine the hypothesis that they have excess polygenic risk. Owing to the high parental history in MODY that may tag inherited polygenic risk, we then performed further analysis adjusting for parental history of diabetes. We performed sensitivity analysis by limiting to each gene and to probands alone. To investigate whether less-deleterious variants are associated with higher polygenic enrichment, we first grouped variants into missense and protein-truncating variants (PTVs), with PTVs assumed to be the most deleterious due to their likely haploinsufficiency effect. We further stratified missense variants by REVEL^59^ (Rare Exome Variant Ensemble Learner) score (<0.9 versus ≥0.9), using it as a proxy for functional severity.

We aimed to include the largest number of MODY cases to maximize the power of the study but were limited by sample and data availability. Based on our final sample size, a post hoc power calculation suggested that we had 80% power to detect minimum differences of 0.08, 0.16 and 0.05 s.d. in polygenic score between controls and genetically confirmed HNF-MODY, unsolved MODY and T2D, respectively. The minimum detectable differences for the clinically referred MODY genetic subgroups were 0.23, 0.16 and 0.094 s.d. for HNF1A, HNF1B and HNF4A, respectively.

#### Assessing impact of common variants on HNF-MODY phenotype

To investigate how common genetic variants influence the clinical presentation of HNF-MODY, we used mixed-effects models to assess associations between PGSs and key outcomes. Specifically, we applied mixed linear models to evaluate the relationship between PGSs and age at diabetes diagnosis, and mixed logistic models to assess associations with diabetes severity. To account for potential within-family correlations that could bias associations, all models included family ID as a random effect. Initial models included all nine polygenic scores to identify independent genetic pathways contributing to variation in clinical presentation. Further analysis focused on scores that were found to be independently associated with modifying the clinical presentation in HNF-MODY, further adjusting for confounding factors that have been previously reported or suspected to influence clinical outcomes. This included sex, age, BMI, year of diabetes diagnosis, proband or family member, variant location, parental history of diabetes (stratified by mother, father or both to capture potential intrauterine exposure), along with the first ten within-cohort principal components. To account for gene-level differences, we included genetic aetiology (MODY gene) as a covariate and examined outcomes separately by gene.

#### Assessing impact of common variants on clinically unselected HNF-MODY carriers

HNF-MODY carriers in the UK Biobank allowed us to assess how common variants affect diabetes risk in a clinically unselected setting. We modelled the probability of diabetes using logistic regression, with T2D PGS as a continuous covariate alongside MODY carrier status and relevant clinical characteristics including sex, age, BMI, parental history of diabetes and the first ten ancestry principal components. Among clinically unselected HNF-MODY carriers, we had 80% power to detect an OR greater than 1.58 per s.d. increase in T2D PGS, below the observed effect size of 2.17. To examine how diabetes risk varies across T2D common variant burden, we computed marginal effects per PGS percentile. Additionally, individuals were stratified into low, intermediate or high PGS groups, defined as the bottom quintile, middle three quintiles and top quintile, respectively, using non-MODY carriers with intermediate T2D risk as the reference group. We used logistic regression to assess differences in diabetes risk relative to the reference group, adjusting for the same covariates.

All statistical analyses were performed using R v.4.4.1 and Stata v.18.

### Reporting summary

Further information on research design is available in the Nature Portfolio Reporting Summary linked to this article.

## Supplementary information

Supplementary Information Supplementary Tables 1–13.

Reporting Summary

## Source data

Source Data Fig. 1 Statistical source data.

Source Data Fig. 2 Statistical source data.

Source Data Fig. 3 Statistical source data.

Source Data Fig. 4 Statistical source data.

Source Data Fig. 5 Statistical source data.

Source Data Extended Data Fig. 1 Statistical source data.

Source Data Extended Data Fig. 2 Statistical source data.

Source Data Extended Data Fig. 3 Statistical source data.

Source Data Extended Data Fig. 4 Statistical source data.

Source Data Extended Data Fig. 5 Statistical source data.

Source Data Extended Data Fig. 6 Statistical source data.

## Data Availability

The data supporting the findings of this study are available within the article, source data and its Supplementary Information. The clinical data, including individual level data, generated and/or analysed as part of this study are not publicly available because of patient confidentiality and ethical approval associated with the data but are available from the corresponding authors upon reasonable request. The UK Biobank dataset is available from https://biobank.ctsu.ox.ac.uk. Source data are provided with this paper.

## Code availability

Publicly available software was used for all analysis, as outlined in the Methods. Code supporting this manuscript is available at https://github.com/ExeterGenetics/teamendo_manuscripts/tree/main/Polygenic_MODY_2025.
