# Supplementary material for: Common genetic variants modify disease risk and clinical presentation in monogenic diabetes
Source: Nat Metab. 2025 Sep 9;7(9):1819–29. doi: 10.1038/s42255-025-01372-0 (PMC12460161; doi:10.1038/s42255-025-01372-0)
Supplement: Supplementary file 1 — Supplementary Tables 1–13. [file 42255_2025_1372_MOESM1_ESM.pdf]

nature metabolism

Supplementary information

https://doi.org/10.1038/s42255-025-01372-0

Common genetic variants modify disease risk and clinical presentation in monogenic diabetes

In the format provided by the authors and unedited

Characteristics	Control	MODY (genetically confirmed mutation in *HNF1A/HNF4A/HNF1B*)	T2D	Unsolved MODY (monogenic diabetes gene panel negative)
N	7,645	1,462	4,773	300
Female Sex, n (%)	4,849 (63.4)	930 (63.6)	1,957 (41)	177 (59)
Diabetes, n (%)	0 (0)	1,462 (100)	4,773 (100)	300 (100)
Age at Recruitment, y	54.3 (15.15)	43.73 (18.29)	68.28 (10.27)	36.55 (12.45)
Age at Diabetes Diagnosis, y	-	22.3 (9.87)	61.99 (11.76)	21.47 (5.88)
BMI (kg/m²)	26.48 (4.68)	25.2 (4.57)	30.83 (5.67)	25.06 (3.14)
Parent Diabetes, n (%)				
None	6,205 (82.9)	197 (14.8)	1,203 (67)	93 (31.1)
Mother	571 (7.6)	609 (45.7)	277 (15.4)	86 (28.8)
Father	621 (8.3)	427 (32)	243 (13.5)	73 (24.4)
Both	84 (1.1)	100 (7.5)	73 (4.1)	47 (15.7)
Insulin Treated, n (%)	0 (0)	539 (40)	325 (11.3)	68 (22.7)
HbA1c, %	5.61 (0.4)	7.64 (1.76)	7.19 (1.12)	8.01 (2.15)

Supplementary Table 1: Characteristics of participants in local cohort at referral for genetic testing. For continuous variables, values are presented as mean (SD), and for categorical variables, counts (n) and percentages (%) are provided. BMI = Body Mass Index, y = years.

Characteristics	HNF1A	HNF1B	HNF4A
N	997	145	320
Female Sex, n (%)	641 (64.3)	76 (52.4)	213 (66.6)
Diabetes, n (%)	997 (100)	145 (100)	320 (100)
Age at Recruitment, y	44.06 (18.67)	36.63 (14.86)	45.91 (17.85)
Age at Diabetes Diagnosis, y	21.66 (9.47)	22.06 (9.58)	24.15 (10.94)
BMI (kg/m²)	25.01 (4.45)	24.64 (5.18)	25.94 (4.89)
Parent Diabetes, n (%)			
None	101 (11)	55 (48.7)	41 (13.7)
Mother	421 (45.7)	37 (32.7)	151 (50.5)
Father	330 (35.8)	16 (14.2)	81 (27.1)
Both	69 (7.5)	5 (4.4)	26 (8.7)
Insulin Treated, n (%)	349 (37.6)	76 (64.4)	114 (38.0)
HbA1c, %	7.56 (1.64)	7.9 (2.44)	7.73 (1.78)

Supplementary Table 2: Characteristics of clinically referred MODY cases by HNF subtype, at time of referral for genetic testing. For continuous variables, values are presented as mean (SD), and for categorical variables, counts (n) and percentages (%) are provided. BMI = Body Mass Index, y = years.

Trait	PubMed ID	N SNPs in Score	Comments
Type 2 Diabetes (T2D)	38374256	1289	Constructed using plink – score function using genome wide significant variants
Type 2 Pathway Scores	38374256	3 - 389	Pathway specific scores of T2D
Type 1 Diabetes (T1D)	30655379	67	Weighted T1D score using T1DGRS2
Acute Insulin Response	28490609	955764	Genome-wide polygenic scores, we implemented the GenoPred 2.2.1 pipeline with LDpred2's auto model, which included quality control of summary statistics and genetic data
Body Mass Index (BMI)	25673413	886707	
Fasting Glucose	34059833	1038695	
Fasting Insulin	34059833	1036765	
HbA1c	34059833	1039883	
Waist Hip Ratio	30239722	906879	
Lipodystrophy	27841877	53	Constructed using plink – score function using genome wide significant variants
Type 2 Diabetes (T2D)*	39379762	1087858	Genome-wide polygenic score for T2D, using weights previously derived using PRS- CS, which excluding UKBB participants during testing

Supplementary Table 3: Polygenic Scores used in analysis. Polygenic scores (PGS) for various traits, including Type 1 and Type 2 Diabetes alongside other diabetes related traits, used in the analysis. For each trait, the corresponding PubMed ID, and the number of SNPs included in the score are provided. * Used in UK Biobank analysis as it does contain have weights from UK Biobank.

PGS	Beta (Kg/m^2)	Lower CI	Upper CI	P Value
T2D	-0.04	-0.32	0.25	8.01E-01
T1D	0.05	-0.23	0.33	7.25E-01
Acute Insulin Response	0.20	-0.09	0.48	1.73E-01
Body Mass Index	1.26	0.98	1.53	1.19E-18
Fasting Glucose	0.01	-0.28	0.28	9.89E-01
HbA1c	-0.16	-0.44	0.12	2.67E-01
Fasting Insulin	0.06	-0.23	0.34	6.98E-01
Lipodystrophy	0.06	-0.23	0.34	7.02E-01
Waist Hip Ratio	-0.04	-0.32	0.25	8.03E-01

Supplementary Table 4: Effect of Polygenic Background on BMI in clinically referred HNF-MODY. Association results between BMI (Kg/m^2) and polygenic scores. Estimates were derived using a mixed-effects linear or logistic model with family as a random effect and adjusted for other polygenic scores, sex, age and the first ten within-cohort principal components.

Predictor		Effect Size (years earlier diabetes diagnosis) (95% CI)	*P*
T2D PGS (per SD increase)		1.01 (0.49 – 1.54)	1.54×10^{-4}
Sex (with respect to females)		2.28 (1.17 – 3.39)	5.56×10^{-5}
BMI (per kg/m^2 increase)		0.24 (0.12 – 0.35)	6.18×10^{-5}
Parent Diabetes History			
	Mother	3.54 (1.89 – 5.17)	2.55×10^{-5}
	Father	0.01 (-1.74 – 1.73)	0.99
	Both	0.01 (2.21 – 2.33)	0.99

Supplementary Table 5: T2D Polygenic lowers age of diagnosis in MODY cases even after adjusting for clinical characteristics. Upper-level polygenic scores identified as independently associated with age of diagnosis in Figure 2A were included in a mixed effect linear regression with age of diabetes as the outcome, and family ID as the random effect. Covariates included in the model: T2D PGS, Sex, BMI, Parental History, Gene, Variant Location, Year of Diabetes diagnosis, Proband/Family Member, and 10 within-cohort principal components. All effect sizes in years. Parental Diabetes in reference to subjects whose parents had no history of diabetes.

Predictor		Odds Ratio (Diabetes Severity) (95% CI)	*P*
T2D PGS (per SD increase)		1.23 (1.06 – 1.43)	0.006
Body Mass Index PGS (per SD increase)		1.31(1.13 -1.52)	4.62×10^{-4}
Sex (with respect to females)		1.05 (0.77 – 1.44)	0.74
BMI (per kg/m^2 increase)		1.07 (1.04 – 1.11)	9.54×10^{-5}
Parent Diabetes History			
	Mother	1.38 (0.85 – 2.23)	0.18
	Father	0.78 (0.47 – 1.29)	0.32
	Both	1.63 (0.83 – 3.22)	0.15

Supplementary Table 6: T2D and BMI Polygenic increase diabetes severity in MODY cases, even after adjusting for clinical characteristics. Upper-level polygenic scores identified as independently associated with diabetes severity in Figure 2B were included in a mixed effect logistic regression with age of diabetes as the outcome, and family ID as the random effect. Covariates included in the model: T2D PGS, Body Mass Index PGS, Sex, BMI, Parental History, Gene, Variant Location, Year of Diabetes diagnosis, Proband/Family Member, and 10 within-cohort principal components. Parental Diabetes in reference to subjects whose parents had no history of diabetes. In total, 676 out of 1462 MODY carriers met the criteria for severe diabetes (defined as HbA1c ≥ 8.5% or insulin treatment at recruitment)

PGS	Gene	Beta (Age Diagnosis)	Lower CI	Upper CI	P Value	Odds Ratio (Severity)	Odds Ratio Lower CI	Odds Ratio Upper CI	P Value
T2D	HNF1A	-1.57	-2.24	-0.90	4.51E-06	1.27	1.04	1.56	2.21E-02
T2D	HNF1B	-0.76	-2.34	0.82	3.43E-01	1.31	0.57	3.04	5.25E-01
T2D	HNF4A	-0.83	-2.12	0.46	2.05E-01	1.09	0.78	1.53	6.17E-01
T1D	HNF1A	0.29	-0.28	0.86	3.21E-01	0.95	0.80	1.12	5.41E-01
T1D	HNF1B	-0.72	-2.31	0.87	3.73E-01	2.38	0.80	7.08	1.19E-01
T1D	HNF4A	0.12	-0.98	1.21	8.32E-01	1.07	0.80	1.44	6.30E-01
Acute Insulin Response	HNF1A	-0.59	-1.21	0.02	5.94E-02	1.03	0.84	1.24	8.01E-01
Acute Insulin Response	HNF1B	-1.87	-3.51	-0.23	2.60E-02	1.16	0.36	3.71	8.07E-01
Acute Insulin Response	HNF4A	0.60	-0.67	1.86	3.53E-01	1.31	0.92	1.87	1.34E-01
Body Mass Index	HNF1A	-0.41	-0.98	0.17	1.64E-01	1.30	1.08	1.57	4.68E-03
Body Mass Index	HNF1B	-0.70	-2.16	0.75	3.41E-01	1.08	0.40	2.91	8.82E-01
Body Mass Index	HNF4A	-0.63	-1.88	0.61	3.17E-01	1.53	1.09	2.16	1.46E-02
Fasting Glucose	HNF1A	-0.60	-1.28	0.08	8.46E-02	1.12	0.91	1.37	3.01E-01
Fasting Glucose	HNF1B	-0.51	-2.64	1.62	6.37E-01	1.26	0.30	5.21	7.52E-01
Fasting Glucose	HNF4A	1.35	-0.03	2.73	5.58E-02	0.94	0.65	1.36	7.27E-01
HbA1c	HNF1A	0.36	-0.25	0.98	2.48E-01	0.94	0.78	1.14	5.34E-01
HbA1c	HNF1B	-0.48	-2.20	1.24	5.82E-01	1.62	0.56	4.69	3.69E-01
HbA1c	HNF4A	-0.30	-1.63	1.03	6.57E-01	1.24	0.86	1.79	2.58E-01
Fasting Insulin	HNF1A	0.23	-0.38	0.85	4.55E-01	1.09	0.90	1.31	3.96E-01
Fasting Insulin	HNF1B	-0.44	-2.29	1.41	6.42E-01	2.23	0.73	6.82	1.58E-01
Fasting Insulin	HNF4A	-0.68	-2.04	0.69	3.29E-01	0.87	0.58	1.29	4.84E-01
Lipodystrophy	HNF1A	0.28	-0.35	0.91	3.81E-01	0.87	0.72	1.05	1.35E-01
Lipodystrophy	HNF1B	1.59	-0.25	3.43	9.06E-02	0.46	0.13	1.58	2.15E-01
Lipodystrophy	HNF4A	1.69	0.38	3.00	1.18E-02	1.12	0.79	1.59	5.23E-01
Waist Hip Ratio	HNF1A	-0.52	-1.13	0.10	1.01E-01	1.00	0.83	1.21	9.72E-01
Waist Hip Ratio	HNF1B	0.44	-1.16	2.04	5.85E-01	2.75	0.90	8.43	7.64E-02
Waist Hip Ratio	HNF4A	-0.36	-1.61	0.90	5.74E-01	0.96	0.68	1.36	8.16E-01

Supplementary Table 7: Effect of Polygenic Score on Phenotype, by MODY subtype. Association results between age of diagnosis (beta) / diabetes severity (odds ratio), split by HNF MODY subtype. Estimates were derived using a mixed-effects linear or logistic model with family as a random effect and adjusted for other polygenic scores and the first ten within-cohort principal components

Predictor	Effect Size (years earlier diabetes diagnosis) (95% CI)	P	Effect Size Birthweight Adjusted (years earlier diabetes diagnosis) (95% CI)	P
T2D PGS (per SD increase)	1.32 (0.30 – 2.33)	0.01	1.31 (0.29 – 2.31)	0.01
Sex (with respect to females)	2.21 (0.08 – 4.34)	0.04	2.24 (0.11 – 4.36)	0.04
BMI (per kg/m^2 increase)	0.06 (-0.13 – 0.25)	0.54	0.07 (-0.11 – 0.27)	0.42
Parent Diabetes History				
Mother	3.70 (0.73 – 6.66)	0.14	3.69 (0.74 – 6.64)	0.14
Father	0.08 (-2.62 – 2.78)	0.95	-0.19 (-2.90 – 2.52)	0.89
Both	0.57 (-3.61 – 4.75)	0.87	0.34 (-3.83 – 4.52)	0.87
Birth Weight (per kg increase)	-	-	-1.0 (-2.2 – 0.2)	0.09

Supplementary Table 8: T2D Polygenic lowers age of diagnosis in MODY, independently of birthweight. Upper-level polygenic scores identified as independently associated with age of diagnosis in Figure 2A were included in a mixed effect linear regression with age of diabetes as the outcome, and family ID as the random effect. To assess the effect of birthweight, models were compared with and without birthweight adjustment in the 413 individuals with available data. Other covariates included in the model: T2D PGS, Sex, BMI, Parental History, Gene, Variant Location, Year of Diabetes diagnosis, Proband/Family Member, and 10 within-cohort principal components. All effect sizes in years. Parental Diabetes in reference to subjects whose parents had no history of diabetes.

Predictor	Odds Ratio (Diabetes Severity) (95% CI)	P	Odds Ratio, Birthweight Adjusted (Diabetes Severity) (95% CI)	P
T2D PGS (per SD increase)	1.34 (1.02 – 1.77)	0.04	1.34 (1.02 – 1.77)	0.04
Body Mass Index PGS (per SD increase)	1.31(0.99 – 1.74)	0.06	1.31(0.99 – 1.74)	0.06
Sex (with respect to females)	0.89 (0.50 – 1.58)	0.70	0.89 (0.51 – 1.57)	0.69
BMI (per kg/m^2 increase)	1.02 (0.97 – 1.08)	0.49	1.01 (0.96 – 1.07)	0.49
Parent Diabetes History				
Mother	0.65 (0.28 – 1.51)	0.32	0.65 (0.28 – 1.50)	0.31
Father	1.27 (0.58 – 2.77)	0.55	1.26 (0.58 – 2.74)	0.57
Both	1.92 (0.61 – 6.11)	0.27	1.90 (0.59 – 6.02)	0.28
Birth Weight (per kg increase)	-	-	1.0 (0.99 - 1.01)	0.97

Supplementary Table 9: T2D PGS increases diabetes severity in MODY cases, independently of birthweight. Upper-level polygenic scores identified as independently associated with diabetes severity in Figure 2B were included in a mixed effect logistic regression with diabetes severity as the outcome, and family ID as the random effect. To assess the effect of birthweight, models were compared with and without birthweight adjustment in the 413 individuals with available data. Other covariates included in the model: T2D PGS, Body Mass Index PGS, Sex, BMI, Parental History, Gene, Variant Location, Year of Diabetes diagnosis, Proband/Family Member, and 10 within-cohort principal components. Parental Diabetes in reference to subjects whose parents had no history of diabetes. In total, 209 out of 413 eligible MODY carriers met the criteria for severe diabetes (defined as HbA1c ≥ 8.5% or insulin treatment at recruitment).

Characteristics		Noncarriers	MODY carriers
N		424,453	100
Female Sex, n (%)		230,412 (54.28)	59 (59%)
Diabetes, n (%)		24,371 (5.74)	49 (49)
Age at Recruitment, y		57.26 (8.03)	57.33 (7.68)
Age at Diabetes Diagnosis, y		59.45 (12.3)	39.01 (17.38)
BMI (kg/m^2)		27.4 (4.75)	26.15 (3.92)
Parent Diabetes, n (%)			
	None	355,550 (83.77)	53 (53)
	Mother	33,069 (7.79)	22 (22)
	Father	31,717 (7.47)	21 (21)
	Both	4,117 (0.97)	≤10
Insulin Treated, n (%)		4,385 (11.58)	14 (27.5)
HbA1c, %		5.64 (0.56)	6.39 (1.11)

Supplementary Table 10: Clinical Characteristics of UK Biobank Participants at Recruitment. Clinical characteristics of UK Biobank participants, split by MODY carriers and noncarriers, at the time of recruitment. For continuous variables, values are presented as mean (SD), and for categorical variables, counts (n) and percentages (%) are provided. BMI = Body Mass Index, y = years.

Gene	Transcript	dna_nomenclature	protein_nomenclature
HNF1A	NM_000545.6	c.1136C>G	p.Pro379Arg
HNF1A	NM_000545.6	c.1309+1G>A	p.?
HNF1A	NM_000545.6	c.1330_1331del	p.Gln444Glufs*104
HNF1A	NM_000545.6	c.1396C>T	p.Gln466*
HNF1A	NM_000545.6	c.1475C>T	p.Thr492Ile
HNF1A	NM_000545.6	c.1487_1494del	p.Leu496Profs*50
HNF1A	NM_000545.6	c.160C>T	p.Arg54*
HNF1A	NM_000545.6	c.343G>T	p.Val115Leu
HNF1A	NM_000545.6	c.347C>T	p.Ala116Val
HNF1A	NM_000545.6	c.391C>T	p.Arg131Trp
HNF1A	NM_000545.6	c.392G>A	p.Arg131Gln
HNF1A	NM_000545.6	c.404del	p.Asp135Valfs*20
HNF1A	NM_000545.6	c.431T>C	p.Leu144Pro
HNF1A	NM_000545.6	c.475C>T	p.Arg159Trp
HNF1A	NM_000545.6	c.526C>T	p.Gln176*
HNF1A	NM_000545.6	c.527-1G>A	p.?
HNF1A	NM_000545.6	c.591G>T	p.Lys197Asn
HNF1A	NM_000545.6	c.598C>T	p.Arg200Trp
HNF1A	NM_000545.6	c.599G>A	p.Arg200Gln
HNF1A	NM_000545.6	c.608G>A	p.Arg203His
HNF1A	NM_000545.6	c.646C>T	p.Gln216*
HNF1A	NM_000545.6	c.685C>T	p.Arg229*
HNF1A	NM_000545.6	c.686G>A	p.Arg229Gln
HNF1A	NM_000545.6	c.812G>A	p.Arg271Gln
HNF1A	NM_000545.6	c.824_826del	p.Glu275del
HNF1B	NM_000458.4	c.1040dup	p.Ser348Valfs*12
HNF1B	NM_000458.4	c.1654-2A>G	p.?
HNF1B	NM_000458.4	c.476C>T	p.Pro159Leu
HNF1B	NM_000458.4	c.493C>T	p.Arg165Cys
HNF1B	NM_000458.4	c.907C>A	p.Arg303Ser
HNF1B	NM_000458.4	17q12 Microdeletion	
HNF4A	NM_175914.4	c.1033G>T	p.Asp345Tyr
HNF4A	NM_175914.4	c.124G>A	p.Gly42Arg
HNF4A	NM_175914.4	c.322G>A	p.Val108Ile
HNF4A	NM_175914.4	c.335G>A	p.Arg112Gln
HNF4A	NM_175914.4	c.352C>T	p.Arg118*
HNF4A	NM_175914.4	c.469A>C	p.Lys157Gln
HNF4A	NM_175914.4	c.530T>C	p.Val177Ala
HNF4A	NM_175914.4	c.537G>A	p.Trp179*
HNF4A	NM_175914.4	c.614A>C	p.His205Pro
HNF4A	NM_175914.4	c.625G>A	p.Gly209Arg
HNF4A	NM_175914.4	c.691C>T	p.Arg231Trp
HNF4A	NM_175914.4	c.733C>T	p.Arg245Cys

HNF4A	NM_175914.4	c.734G>A	p.Arg245His
HNF4A	NM_175914.4	c.787G>C	p.Glu263Gln
HNF4A	NM_175914.4	c.823C>T	p.Pro275Ser
HNF4A	NM_175914.4	c.869G>A	p.Arg290His
HNF4A	NM_175914.4	c.925C>T	p.Arg309Cys
HNF4A	NM_175914.4	c.926G>A	p.Arg309His
HNF4A	NM_175914.4	c.932G>A	p.Arg311His
HNF4A	NM_175914.4	c.956T>C	p.Leu319Pro

Supplementary Table 11: Pathogenic variants of *HNF1A*, *HNF1B* and *HNF4A* identified in the UK Biobank

Group	Prevalence	h2 (GCTA-GREML-LDMS)	SE (GCTA-GREML-LDMS)	P (GCTA-GREML-LDMS)	h2 (LDAK-REML)	SE (LDAK-REML)	P (LDAK-REML)	h2 (LDAK-PCGC)	SE (LDAK-PCGC)	P (LDAK-PCGC)
All HNF MODY	0.0005	0.239224	0.034465	1.50E-06	0.228802	0.033119	6.45E-14	0.220527	0.10972	5.41E-03
All HNF MODY	0.00025	0.217268	0.031302	1.50E-06	0.207506	0.030037	6.45E-14	0.202287	0.101634	5.94E-03
HNF1A MODY	0.0005	0.270959	0.052318	1.28E-04	0.255482	0.050068	4.29E-08	0.321433	0.191599	1.63E-02
HNF1A MODY	0.00025	0.24609	0.047516	1.28E-04	0.231703	0.045408	4.29E-08	0.29589	0.177519	1.72E-02
T2D	0.1	0.30841	0.029422	1.47E-07	0.290519	0.028217	9.81E-28	0.278013	0.042226	4.36E-26

Supplementary Table 12: SNP based heritability estimates for MODY and T2D. Common variant heritability (h2) was estimated on the liability scale (i.e. across various disease prevalences). Estimates were obtained using GCTA-GREML, with LDAK-REML and LDAK-PCGC for sensitivity analysis, and also sub setting to carriers of *HNF1A* MODY. Heritability analysis was restricted to unrelated individuals.

Characteristics	MODY	Unsolved MODY	P Value
N	1,462	300	-
Female Sex, n (%)	930 (63.6)	177 (59)	0.13
Diabetes, n (%)	1,462 (100)	300 (100)	1
Age at Recruitment, y	43.73 (18.29)	36.55 (12.45)	1.1×10^{-10}
Age at Diabetes Diagnosis, y	22.3 (9.87)	21.47 (5.88)	0.07
BMI (kg/m²)	25.2 (4.57)	25.06 (3.14)	0.63
Parent Diabetes, n (%)			2.2×10^{-16}
None	197 (14.8)	93 (31.1)	
Mother	609 (45.7)	86 (28.8)	
Father	427 (32)	73 (24.4)	
Both	100 (7.5)	47 (15.7)	
Insulin Treated, n (%)	539 (40)	68 (22.7)	2×10^{-8}
HbA1c, %	7.64 (1.76)	8.01 (2.15)	0.0037

Supplementary Table 13: Comparison of clinical features, collected at referral for genetic testing, between genotype-positive and genotype-negative (Unsolved) MODY cases. Continuous variables were assessed using t-tests, and categorical variables were assessed using chi-square tests. For continuous variables, values are presented as mean (SD). For categorical variables, counts (n) and percentages (%) are provided. BMI = Body Mass Index, y = years.
