# Supplementary material for: Common genetic variants modify disease risk and clinical presentation in monogenic diabetes
Source: Nat Metab. 2025 Sep 9;7(9):1819–29. doi: 10.1038/s42255-025-01372-0 (PMC12460161; doi:10.1038/s42255-025-01372-0)
Supplement: Supplementary file 2 — Reporting Summary [file 42255_2025_1372_MOESM2_ESM.pdf]

nature portfolio

Corresponding author(s): Kashyap Patel

Last updated by author(s): Aug 4, 2025

Reporting Summary

Nature Portfolio wishes to improve the reproducibility of the work that we publish. This form provides structure for consistency and transparency in reporting. For further information on Nature Portfolio policies, see our Editorial Policies and the Editorial Policy Checklist.

Statistics

For all statistical analyses, confirm that the following items are present in the figure legend, table legend, main text, or Methods section.

n/a	Confirmed	
☐	☒	The exact sample size (*n*) for each experimental group/condition, given as a discrete number and unit of measurement
☒	☐	A statement on whether measurements were taken from distinct samples or whether the same sample was measured repeatedly
☐	☒	The statistical test(s) used AND whether they are one- or two-sided<br>*Only common tests should be described solely by name; describe more complex techniques in the Methods section.*
☐	☒	A description of all covariates tested
☐	☒	A description of any assumptions or corrections, such as tests of normality and adjustment for multiple comparisons
☐	☒	A full description of the statistical parameters including central tendency (e.g. means) or other basic estimates (e.g. regression coefficient) AND variation (e.g. standard deviation) or associated estimates of uncertainty (e.g. confidence intervals)
☐	☒	For null hypothesis testing, the test statistic (e.g. F, t, r) with confidence intervals, effect sizes, degrees of freedom and P value noted<br>*Give P values as exact values whenever suitable.*
☒	☐	For Bayesian analysis, information on the choice of priors and Markov chain Monte Carlo settings
☐	☒	For hierarchical and complex designs, identification of the appropriate level for tests and full reporting of outcomes
☐	☒	Estimates of effect sizes (e.g. Cohen's *d*, Pearson's *r*), indicating how they were calculated

Our web collection on statistics for biologists contains articles on many of the points above.

Software and code

Policy information about availability of computer code

Data collection	No Software was used for data collection
Data analysis	In this analysis we used publicly available software including: Genopred (v2.2.1), KING robust algorithim (v2.2.4), FlashPCA(v2.0), PLINK (v1.9), GCTA (v1.94.1) LDAK (v6.0), R (v4.4.1) and Stata (v18.0). Further detail is provided in the Methods Section.

For manuscripts utilizing custom algorithms or software that are central to the research but not yet described in published literature, software must be made available to editors and reviewers. We strongly encourage code deposition in a community repository (e.g. GitHub). See the Nature Portfolio guidelines for submitting code & software for further information.

Data

Policy information about availability of data

All manuscripts must include a data availability statement. This statement should provide the following information, where applicable:

- Accession codes, unique identifiers, or web links for publicly available datasets
- A description of any restrictions on data availability
- For clinical datasets or third party data, please ensure that the statement adheres to our policy

The data supporting the findings of this study are available within the article, source data, and its supplemental information. The clinical data, including individual level data, generated and/or analysed as part of this study are not publicly available because of patient confidentiality and ethical approval associated with the data but are available from the corresponding authors upon reasonable request. The UK Biobank dataset is available from https://biobank.ctsu.ox.ac.uk

Research involving human participants, their data, or biological material

Policy information about studies with human participants or human data. See also policy information about sex, gender (identity/presentation), and sexual orientation and race, ethnicity and racism.

Reporting on sex and gender	Information on sex was collected at patient recruitment to the UK Biobank or for genetic testing for our local cohorts. We removed participants when their sex inferred by genetic data did not match reported data. We performed analysis with both sex combined, correcting for sex as a covariate. Sample sizes by sex can be found in supplementary tables.
Reporting on race, ethnicity, or other socially relevant groupings	We only used individuals who had an inferred European ancestry, defined in the methods section. We also used genetic and/or within cohort principal components to further adjust for population structure
Population characteristics	We have outlined the population characteristics for our cohorts in detail in the methods section, with more detail contained in the supplementary tables. Population characteristics e.g.. sex, age, parental history of diabetes. were included as covariates when appropriate.
Recruitment	In our local MODY cohort we analysed individuals referred for monogenic diabetes genetic testing at the Exeter Genomics Laboratory, Royal Devon University Healthcare NHS Foundation Trust, Exeter, UK. These referrals originated from clinical suspicion of MODY during routine clinical care in the UK. We also analysed unselected T2D and non diabetic participants from two ethically approved population cohorts in Southwest England: The Exeter 10000 study and The Diabetes Alliance for Research in England (DARE) Study. For non-clinically referred individuals, recruitment was performed by UK Biobank, between 2006 and 2010.
Ethics oversight	This study complies with all relevant ethical regulation and was approved by the appropriate ethics committees. Our study combined three ethically approved cohorts. In our local MODY cohort, all probands or their guardians provided informed consent, and the North Wales Ethics Committee approved the study, with Genetic Beta Cell Research Bank approving sample access. The NIHR Exeter Clinical Research Facility management committee approved access to these samples and genotype data for our T2D and non-diabetic controls. This research also utilised data from the UK Biobank resource carried out under UK Biobank application number 103356. UK Biobank protocols were approved by the National Research Ethics Service Committee.

Note that full information on the approval of the study protocol must also be provided in the manuscript.

Field-specific reporting

Please select the one below that is the best fit for your research. If you are not sure, read the appropriate sections before making your selection.

☒ Life sciences ☐ Behavioural & social sciences ☐ Ecological, evolutionary & environmental sciences

For a reference copy of the document with all sections, see nature.com/documents/nr-reporting-summary-flat.pdf

Life sciences study design

All studies must disclose on these points even when the disclosure is negative.

Sample size	Sample Size is reported in the manuscript and with further detail in supplementary tables. In the UK Biobank we included 424,553 individuals who had both exome and array data and were of genetically inferred European ancestry.
Data exclusions	In this study we excluded individuals who were not of genetically inferred European ancestry. We also applied detailed sample quality quality control, such as removing sex mismatches, from our local array data. Quality control on UK Biobank data is provided in text and in refferenced publications.
Replication	We demonstrate the effect of polygenic effects in MODY in two cohorts. Clinically referred MODY patients and clinically unselected MODY carriers in the UK Biobank.
Randomization	Not relevant in this study design
Blinding	Not relevant in this study design

Reporting for specific materials, systems and methods

We require information from authors about some types of materials, experimental systems and methods used in many studies. Here, indicate whether each material, system or method listed is relevant to your study. If you are not sure if a list item applies to your research, read the appropriate section before selecting a response.

Materials & experimental systems

n/a	Involved in the study
☒	☐ Antibodies
☒	☐ Eukaryotic cell lines
☒	☐ Palaeontology and archaeology
☒	☐ Animals and other organisms
☒	☐ Clinical data
☒	☐ Dual use research of concern
☒	☐ Plants

Methods

n/a	Involved in the study
☒	☐ ChIP-seq
☒	☐ Flow cytometry
☒	☐ MRI-based neuroimaging

Plants

Seed stocks	NA
Novel plant genotypes	NA
Authentication	NA
